# Reorganization

**Published:** 1897-04

**Authors:** 


### Editorial.

### REORGANIZATION.

UNDER the heading of Original Communications will be found in the present number a very suggestive article by Professor Fillebrown, chairman of the committee appointed by the American Dental Association, in 1894, to take into consideration the subject of the union of the American with the Southern Association. While this article does not claim to embody all the thoughts of the joint committees, it evidently may be regarded as voicing the general sentiment, and it is, therefore, especially worthy of serious thought.

It is eminently proper before making a final report to the meetings to be held at Old Point Comfort, in August next, that the committee should formulate a preliminary plan in order that the subject may be crystallized in the minds of the dental profession throughout the country, and thus be prepared for the discussion of the topic in all sections, and enable those who expect to be present at Old Point Comfort to bring with them their best and most matured reflections.

The change proposed is a radical one, and will require, and doubtless will receive, careful consideration. That it will invite opposition is more than probable, for old attachments cannot be uprooted in an hour or even in years, but, as we are obliged to part at some period with our loves and hopes in this unstable life of ours, it may be that the time has arrived for the death of the old in associative effort and the birth of a more active body.

It has been apparent for a long time, as intimated in a former article on this subject, that present methods have outlived their usefulness; that the dental profession was wasting its efforts, with weakened results, in lack of concentration. Society after society has been formed and combinations of societies have been organized to cover certain districts to an extent that diminishes power, while, at the same time, it increases the demand for papers which the limited range of thought and practice in dentistry will not and cannot supply.

With this fact in view, it certainly becomes each interested member of the dental profession of this part of the world to calmly consider whether it may not be better to make sacrifices of feeling and even bury old loves that the younger generation may open the coming century with a more vigorous associative life.

The writer sympathizes with the old feeling, and when this idea of the committee was first brought to his attention it was met with a positive degree of repugnance, but a calmer consideration leads, if not to acceptance, at least to a willingness to accord to it recognition and judicial examination that in the end all may become more thoroughly united in our work.

The details as worked out in the article of Professor Fillebrown cannot be considered at this time. The majority of them are valuable and some of them are objectionable in a new organization, but these will come up for consideration in the future. The main point is to have all workers in the dental ranks think over the subject and be prepared to act.

There is one excellent feature in a new organization that must recommend it to the young, and that is that a body such as proposed offers an opportunity for a new and more vigorous element to enter and take part in its deliberations. There is nothing more difficult for young persons than to enter as members in an old organization and attempt an active participation in its work. The old members too frequently regard their presence as not an element of strength, and the young feel the crushing effect of age and experience. For a time, at least, all, young and old, would be practically upon the same level, and thus an opportunity would be given for the infusion of new life and more energetic methods.

There is one thing which, in the opinion of the writer, should be avoided, and that is a subserviency to old methods of organization. We have as a branch of the medical profession followed too long its plans, as though nothing could be evolved of a better character. When it became necessary for the more perfect arrangement of college work, a few stepped aside from old ruts and organized, with some degree of timidity, the National Association of Dental Faculties. That was truly an original conception, and the success attained warranted the effort, for it not only has advanced the standard of dental education, but has made a path for the medical colleges to follow.

This example should be an incentive to try and organize a distinctively original body, and not to copy old lines entirely, as the suggestions seem to indicate as the purpose of the committee.

It will take time to complete the organizations proposed, and probably the new century will have opened before even the central association, if decided upon, will have been properly in complete working order, but better delay than that any mistake should be made.

It is hoped that all societies will send strong delegations down to Old Point Comfort, and in order that these may be intelligent upon the subject, the matter should be given time for thorough discussion in all dental bodies throughout the length and breadth of the land, for it is only by this interchange of thought that the right course to pursue can intelligently be marked out.

